# Chronodisruption that dampens output of the central clock abolishes rhythms in metabolome profiles and elevates acylcarnitine levels in the liver of female rats

**DOI:** 10.1111/apha.14278

**Published:** 2025-01-13

**Authors:** Shiyana Arora, Pavel Houdek, Tomáš Čajka, Tereza Dočkal, Martin Sládek, Alena Sumová

**Affiliations:** ^1^ Laboratory of Biological Rhythms Institute of Physiology of the Czech Academy of Sciences Prague Czech Republic; ^2^ Laboratory of Translational Metabolism Institute of Physiology of the Czech Academy of Sciences Prague Czech Republic

**Keywords:** acylcarnitine, chronodisruption, clock, female, glucose homeostasis, liver, metabolome, pancreas, rat, sleep, suprachiasmatic nucleus

## Abstract

**Aim:**

Exposure to light at night and meal time misaligned with the light/dark (LD) cycle—typical features of daily life in modern 24/7 society—are associated with negative effects on health. To understand the mechanism, we developed a novel protocol of complex chronodisruption (CD) in which we exposed female rats to four weekly cycles consisting of 5‐day intervals of constant light and 2‐day intervals of food access restricted to the light phase of the 12:12 LD cycle.

**Methods:**

We examined the effects of CD on behavior, estrous cycle, sleep patterns, glucose homeostasis and profiles of clock‐ and metabolism‐related gene expression (using RT qPCR) and liver metabolome and lipidome (using untargeted metabolomic and lipidomic profiling).

**Results:**

CD attenuated the rhythmic output of the central clock in the suprachiasmatic nucleus via *Prok2* signaling, thereby disrupting locomotor activity, the estrous cycle, sleep patterns, and mutual phase relationship between the central and peripheral clocks. In the periphery, CD abolished *Per1,2* expression rhythms in peripheral tissues (liver, pancreas, colon) and worsened glucose homeostasis. In the liver, it impaired the expression of NAD^+^, lipid, and cholesterol metabolism genes and abolished most of the high‐amplitude rhythms of lipids and polar metabolites. Interestingly, CD abolished the circadian rhythm of *Cpt1a* expression and increased the levels of long‐chain acylcarnitines (ACar 18:2, ACar 16:0), indicating enhanced fatty acid oxidation in mitochondria.

**Conclusion:**

Our data show the widespread effects of CD on metabolism and point to ACars as biomarkers for CD due to misaligned sleep and feeding patterns.

## 1 INTRODUCTION

Modern society depends on shift workers who are exposed to periods of light and meals at night with the schedules alternating during the working days and the off‐days. Even people working standard hours often expose themselves to light during the night and change their eating habits accordingly. Together with insufficient exposure to natural daylight, this leads to improper entrainment of circadian system with a solar day. Most people with such lifestyle tend to shift their activity/eating into nighttime during the off‐work days, manifested as late chronotype,^1^, ^2^ which has been associated with impaired health, represented not only by neuropsychiatric,^3^ but also cardiovascular and metabolic^4^, ^5^ disorders. The mechanisms underlying these effects have not been fully ascertained.

The circadian system is comprised of the central clock in the suprachiasmatic nuclei of the hypothalamus (SCN),^6^, ^7^ and the peripheral clocks in other tissues and cells in the body.^8^ The circadian signal is generated autonomously^9^ by a mechanism based on coordinated transcriptional‐translational feedback loops (TTFLs) driving rhythmic expression of clock genes which are coupled with cycles in posttranslational modifications of the clock proteins (for review, see^10^). The mechanism primarily comprises of transcriptional activators CLOCK and BMAL1 which bind promoters of genes coding repressors PER1‐3 and CRY1,2. Additional loop utilizes activators RORs and repressors NR1D1,2, which drive rhythm in expression of *Bmal1* gene. Importantly, the circadian signal is spread onto various cellular, tissue and systemic processes via rhythmical binding of clock proteins to promoters of other genes that rhythmically activates (CLOCK::BMAL1) or inhibits (NR1D1) their transcription. The TTFLs operate with a period that deviates from the 24 hours, therefore, to run in sync with the solar day, the clocks need to be “entrained,” which means their pace and phase needs to be adjusted according to the environmental cycle.^9^ The entrainment involves stimulus‐specific modifications (activation or suppression) of expression of the clock genes or proteins that lead to a change in stoichiometry of the TTFL.^11^

The regular light/dark (LD) cycle is the most powerful environmental cue for entrainment of the SCN clock,^12^, ^13^ whereas feeding/fasting cycle is one of the potent entraining cues for the peripheral clocks.^14^, ^15^, ^16^, ^17^, ^18^ After the SCN clock entrains to external LD cycle, it drives multiple systemic rhythms, e.g., in the activity, food intake, hormonal levels, body temperature, etc., that entrain peripheral clocks accordingly.^19^ The mechanism involves clock‐driven rhythms in the release of various neurohumoral factors,^9^ whose genes are under the direct control of the TTFL, such as *arginine‐vasopressin* (*Avp*), *vasoactive intestinal polypeptide* (*Vip*) or *prokineticin 2* (*Prok2*). They mediate the rhythmic signals to the brains regions that affect neural and humoral regulatory pathways that entrain the peripheral clocks.^20^, ^21^, ^22^ The phases of the peripheral clocks are thus lagging the central clock by several hours.^15^, ^23^

For light entrainment of the SCN clock, mostly activation of *Per1* expression is involved.^24^ In peripheral clocks, *Per2* is under the control of the local peripheral oscillator and, at the same time, is sensing the systemic signals from the SCN clock.^25^ Receiving these signals is important to keep internal synchrony among the clocks in the body. It is believed that disruption of the internal synchrony, referred to as chronodisruption, may contribute to pathology via disturbance of circadian control of the wide array of cellular and tissue‐specific processes, documented by findings that high percentage of transcriptome,^26^, ^27^ proteome^28^, ^29^ and metabolome^26^, ^30^ is under control of the clock. In animal research, the long‐term chronodisruption has been associated with a higher incidence of cancer,^31^ metabolic disorders,^32^, ^33^ neurodegeneration,^34^ and others.

Most of the animal studies on chronodisruption focused on analyzing the impact of either reversal in feeding regime,^35^, ^36^ shift in LD cycles,^37^, ^38^ or constant light.^38^, ^39^ Whereas the SCN clock is significantly dampened in constant light,^38^, ^40^, ^41^ it does not respond to changes in feeding/fasting cycle under the standard LD conditions.^42^ Nevertheless, SCN becomes partially sensitive to feeding regime under specific conditions when the strength of the signal is critical, like under caloric restriction,^43^ or when the clock is weakened, like under constant light.^44^ In contrast, clocks in several peripheral tissues, namely liver,^42^ pancreas,^45^ colon,^39^ heart,^42^ etc., are dominantly sensitive to changes in feeding regime over the SCN signals. Additionally, they differ in their persistence under constant light and their re‐establishment by the restricted feeding.^39^ Although human behavior in real life often affects the integrity of the entire circadian system, the effects of long‐term chronodisruption are not well understood. One of the reasons for this is the lack of animal models that simulate the complexity of chronodisruption. We hypothesized that misalignment of feeding patterns to LD cycles combined with an intermittent attenuation of entrainment with LD cycles causes a complex chronodisruption leading to an impaired metabolic state. Therefore, we developed a novel protocol for chronodisruption that combines attenuation of SCN signaling (by exposing animals to constant light for 5 days) with episodes of a misaligned feeding regime (via providing food only during the light phase of the LD cycle for 2 days), repeated in 4 weekly cycles. We used female rats as a model because (a) females are typically more sensitive to chronodisruption than males as shown in human population studies,^5^, ^46^ and (b) rat circadian system resembles that in humans more than that of mouse because of intact melatonin production.^47^

## 2 RESULTS

### 2.1 CD affects spontaneous locomotor activity, estrous cycle, and related sleep patterns

**FIGURE 1:**
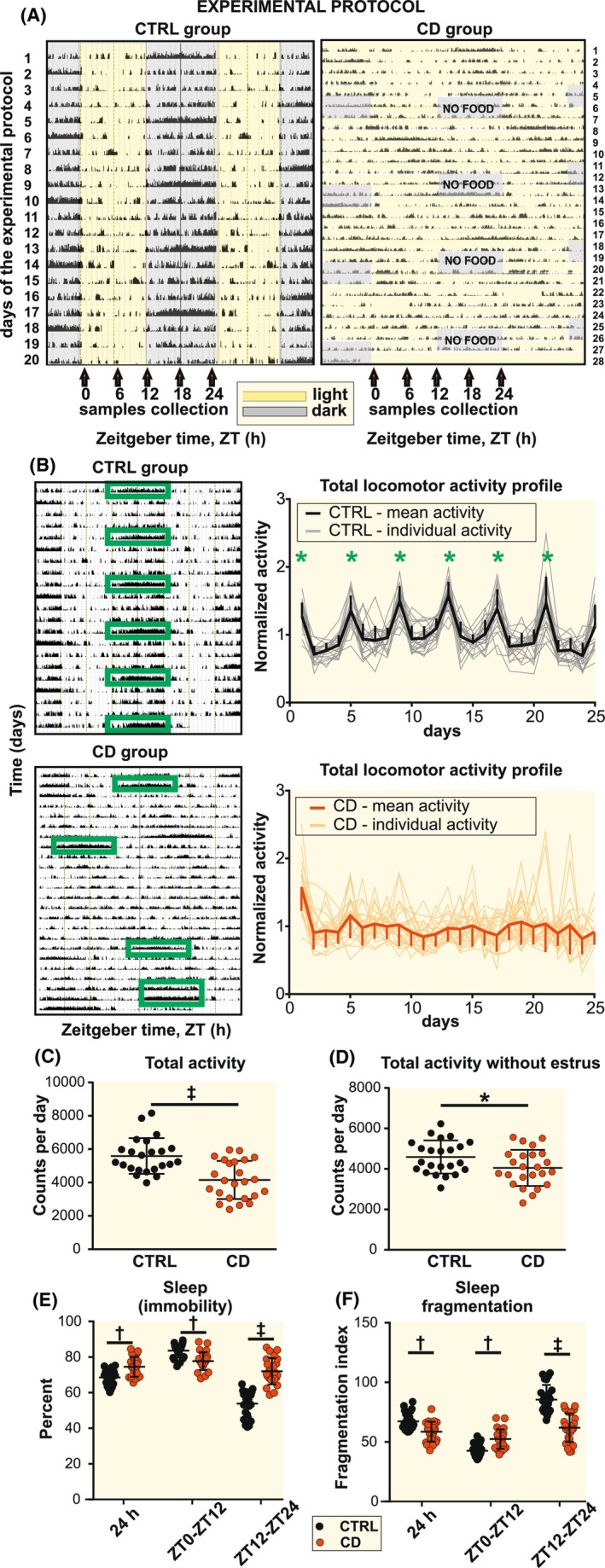
Circadian activity, estrous cycle, and sleep are disrupted by CD. (A) Experimental protocol showing representative double‐plotted actogram of a rat from control (CTRL, left) and chronodisruption (CD, right) group. Yellow shading depicts the time with lights on (Zeitgeber time 0), gray depicts lights off (Zeitgeber time 12). The animals had ad libitum access to food with the exception of the lights off interval in the CD group (gray + NO FOOD). Arrows below the actogram show the time of sampling tissues at the end of the experiment. (B) Representative actograms (left) and mean activity profiles of all rats from CTRL (up) or CD (down) groups with green rectangles and green stars depicting increased activity during estrus phase. Notice the robust disruption of a regular 4‐day estrous cycle patterns in females under CD. (C) Total locomotor activity was significantly decreased under CD (*t*‐test, *p* < 0.0001), (D) even when calculated without the hyperactivity during estrus (*p* = 0.0375). Sleep (percent of immobility of the total individual activity of each rat) (E) and sleep fragmentation index (F) were significantly (two‐way ANOVA with Sidak's test) affected by CD depending on the time of day. Individual data, as well as mean ± SD, are shown (CTRL, *n* = 23; CD, *n* = 26). *p < 0.05, †*p* < 0.01, ‡*p* < 0.0001.

The representative activity records of rats from CTRL and CD groups are shown in Figure 1A (for all records, see Figure S1). Rats of the CTRL group maintained in LD12:12 and fed ad libitum were active mostly during darkness, as expected. There was an apparent effect of estrous cycle on the locomotor activity with significant hyperactivity on the day of estrus, repeating with a regularity of 4 days over the experiment (Figure 1B, upper graph). Rats of the CD group exposed to 4 weekly cycles each comprised of constant light (5 days) and reversed feeding on LD12:12 (2 days) exhibited variable patterns of activity profiles; most of the rats were almost arrhythmic (approximately 2/3) and the rest maintained weak rhythms with a long period (25.47 ± 2.28, *n* = 24). In the CD group, the 4‐day rhythm in estrous cycle‐driven hyperactivity was disrupted (days with hyperactivity occurred randomly) or completely abolished (Figure 1B, lower graph). The total activity was significantly lower in the CD group (*t*‐test, *p* < 0.0001) when calculated for all days of the experimental protocol (Figure 1C), and the difference was smaller but still significant if the days with the estrous cycle‐induced hyperactivity were excluded from the analysis (*t*‐test, *p* = 0.0375) (Figure 1D).

To assess the effect of CD on the sleep parameters, data were analyzed for duration of immobility as percent of total activity of each animal (as a proxy of time spent sleeping) (Figure 1E) and fragmentation index (as a marker of sleep bouts frequency) (Figure 1F). The factor of estrous cycle was not included into this analysis. In accordance with the measurement of total activity (Figure 1B), CD rats had higher percent of immobility over 24 h (two‐way ANOVA, *p* < 0.0015), which indicates that they were in general less active. The CD group was slightly more active (lower immobility index) than the CTRL group during the light phase (ZT0–ZT12) (two‐way ANOVA, *p* < 0.0021), but less active (higher immobility index) during the dark phase (ZT12–ZT24) (two‐way ANOVA, *p* < 0.0001). Furthermore, CD rats had lower fragmentation index over 24 h compared to CTRL rats (two‐way ANOVA, *p* = 0.0056), which was due to more consolidated (less fragmented) sleep during the dark phase (ZT12–ZT24) (two‐way ANOVA, *p* < 0.0001), whereas more fragmented sleep during the light phase (ZT0–ZT12) (two‐way ANOVA, *p* = 0.0013), Therefore, the CD significantly increased and consolidated time when the rats rested during the darkness (active part of the day) and decreased and fragmented time when they rested during the light phase (sleep time).

The results show that CD suppressed total activity, disrupted estrous cycle, and changed temporal sleep patterns.

### 2.2 CD affects glucose tolerance and food intake

**FIGURE 2:**
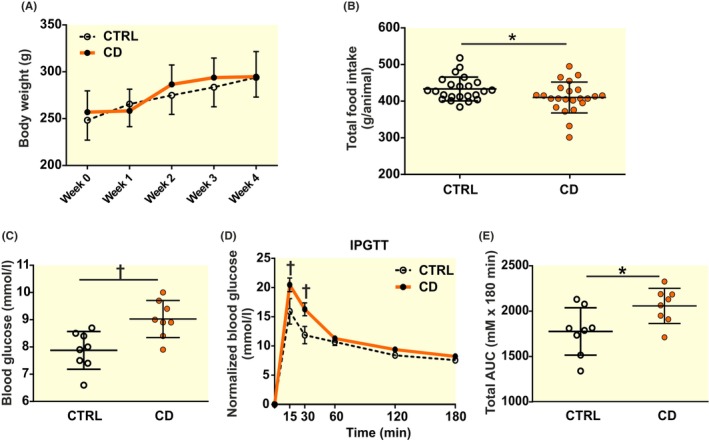
CD affects glucose metabolism. (A) While the animal body weight remained similar in both groups, food intake (B) was slightly lower (*t*‐test, *p* = 0.04) in CD group, and blood glucose (C) analyzed by glucose meter from the tail vein was significantly higher (*t*‐test, *p* = 0.0047) in CD group. (D) Intraperitoneal glucose tolerance test (IPGTT) showed significantly slower clearance of injected glucose load (elevated levels 15 and 30 min after the glucose injection, two‐way ANOVA with Sidak's test, *p* = 0.0055 and *p* = 0.0075, respectively) (E) larger overall area under curve (AUC, *t*‐test, *p* = 0.028). *p < 0.05, †*p* < 0.01

Body weight (BW), which was monitored in all animals every week of the experiment, did not differ between CTRL and CD animals (two‐way ANOVA, *p* = 0.10) (Figure 2A), although total amount of food that animals of the CD group consumed (Figure 2B) was slightly lower (*t*‐test, *p* = 0.040), likely as a consequence of the 2‐day rRF in the protocol. Despite that, basal blood glucose levels at the end of the protocol (Figure 2C) were significantly higher (*t*‐test, *p* = 0.0047) in CD (*n* = 8; BW 292 ± 19 g) than in CTRL (n = 8; BW 294 ± 11 g) group. Furthermore, the results of IPGTT performed during the last week of the experimental protocol (n = 8 per group) showed worsening in glucose compensation due to CD; in the CD group the blood glucose levels detected 15 and 30 min after the glucose injection (two‐way ANOVA, *p* = 0.0055 and *p* = 0.0075, respectively) (Figure 2D) as well as the total AUC value (*t*‐test, *p* = 0.028) (Figure 2E) were significantly higher compared to CTRL group. The data show that despite decreased total food consumption and no effect on BW, the CD significantly impaired glucose homeostasis.

### 2.3 CD affects expression of selective clock and clock‐controlled genes in the SCN

**FIGURE 3:**
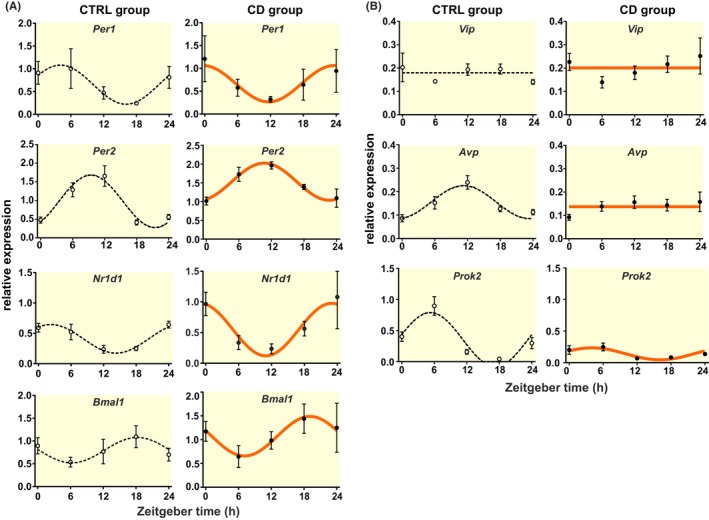
Daily mRNA profiles of selected clock genes (A) and neuropeptide genes (B) in the suprachiasmatic nucleus (SCN) of the hypothalamus measured by RT qPCR in CTRL (black dashed line) and CD (orange full line) group and analyzed by cosinor; mean ± SEM.

To ascertain the effect of our complex chronodisruption protocol on the circadian system, we first assessed expression of four canonical clock genes (*Per1*, *Per2*, *Nr1d1*, and *Bmal1*) and three clock‐output related genes (*Vip*, *Avp*, and *Prok2*) in the SCN of CTRL and CD animals. We found that in the CTRL group, expression of all clock genes exhibited circadian rhythms with phases as expected according to the TTFL model (Figure 3A; Table S2 for results of cosinor analysis). Exposure of rats to CD protocol did not abolish rhythmicity in the expression of clock genes (Figure 3A; Table S2 for results of cosinor analysis). We consider the small differences in acrophases of these rhythms non‐significant based on the 6‐h intervals of the sample collection. In the SCN of CTRL group, expression of clock‐controlled genes *Avp* and *Prok2* exhibited significant circadian rhythms (Figure 3B) in accordance with previously published data.^20^, ^48^ Importantly, exposure to CD either abolished or severely dampened *Avp* and *Prok2* expression rhythms, respectively; *Vip* mRNA profile, which was not rhythmic in the CTRL group, was not affected by CD (Figure 3B; Table S3). The results show that CD significantly dampened rhythmic output generated by the SCN clock via dysregulation of neurohumoral signaling.

### 2.4 CD affects daily profiles of clock gene expression in the peripheral clocks in a gene‐specific manner

To ascertain how disturbance of the SCN rhythmic output affected clock in the periphery, we assessed daily mRNA profiles of clock genes (*Per1*, *Per2*, *Nr1d1*, and *Bmal1*) in the brain (DMH) and various tissues (liver, colon, and pancreas) of CTRL and CD animals.

**FIGURE 4:**
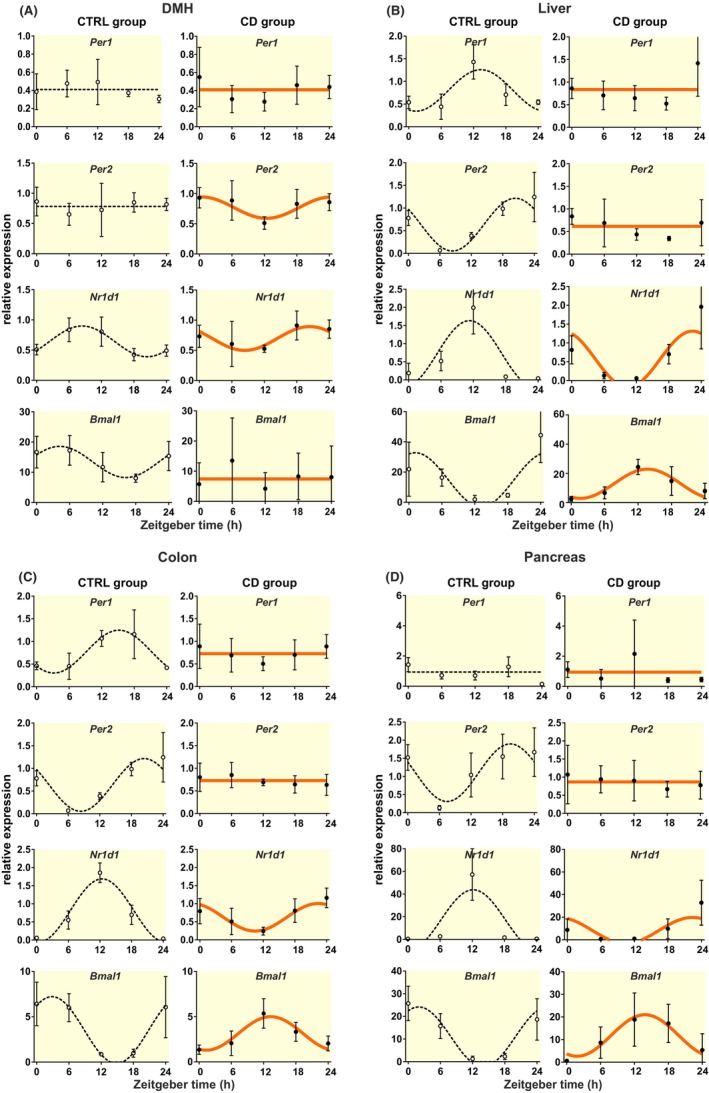
Daily mRNA profiles of selected clock genes in the (A) dorsomedial hypothalamus (DMH), (B) liver, (C) colon, and (D) pancreas, measured by RT qPCR in CTRL (black dashed line) and CD (orange full line) group and analyzed by cosinor; mean ± SEM.

**FIGURE 5:**
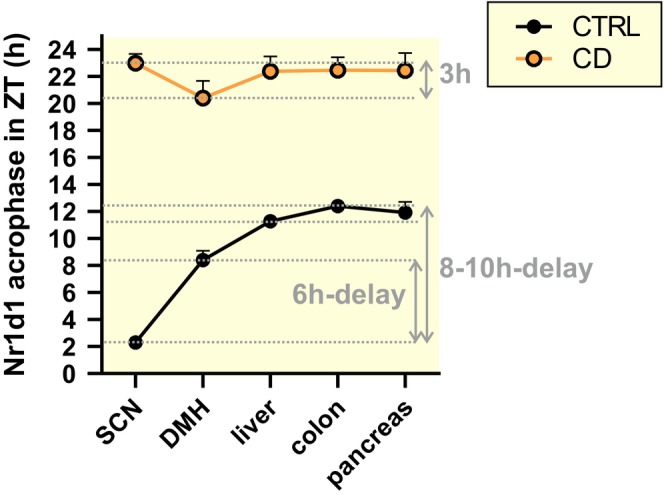
Circadian phase (acrophase) of *Nr1d1* (*Rev‐Erbα*) mRNA in the SCN, DMH and peripheral tissues (see the original data in Figures 4 and 5 and Tables S2 and S3) from CTRL (black) and CD (orange) group calculated by cosinor. Notice the effect of CD on phase relationship between the SCN and periphery.

In DMH of the CTRL group, circadian rhythms in the expression of *Per1* and *Per2* genes were not confirmed, but expression of *Nr1d1* and *Bmal1* exhibited shallow rhythms (Figure 4A, Table S2) with a phase delayed relatively to the SCN clock as expected (Figure 5). Exposure to CD induced a weak but significant circadian rhythm in *Per2*, reversed the phase of the *Nr1d1*, and completely abolished the *Bmal1* rhythm (Figure 4A, Table S2).

The liver, colon, and pancreas exhibited robust rhythmicity of all studied clock genes in the CTRL group as expected. They responded to the CD similarly, that is, both *Per* genes lost the rhythms, and *Nr1d1* and *Bmal1* rhythms phase reversed and their amplitudes were suppressed (Figure 4B–D, Table S2). The results show that circadian oscillators in the DMH, liver, colon and pancreas respond to CD in a tissue‐ and gene‐specific manner. Comparison of phases of the clocks in the individual tissues (via *Nr1d1* expression profiles, which is the only gene rhythmic in all studied tissue samples and both experimental conditions) revealed that CD‐dampened SCN output resulted in abolishment of the phase‐leading position of the SCN clock relative to the peripheral clocks, as typical under the control conditions. Instead, phases of all clocks became more or less aligned, peaking between ZT20 and ZT24 (Figure 5, for data see Table S2).

### 2.5 CD affects daily metabolomic and lipidomic profiles

Since our data showed that CD altered the expression of clock genes in the liver and significantly slowed the glucose clearance rate, suggesting a disruption of glucose metabolism, we decided to investigate the effects of CD on liver metabolism at the molecular level. To that end, we conducted untargeted metabolomic and lipidomic profiling of liver samples collected from rats of the CTRL and CD groups at 5 different time points (ZT0, ZT6, ZT12, ZT18, and ZT24; *n* = 4‐5/time point). We used reversed‐phase liquid chromatography–mass spectrometry (RPLC–MS) in positive and negative ion mode for complex lipids followed by analysis of polar metabolites using RPLC–MS and hydrophilic interaction chromatography–mass spectrometry (HILIC‐MS), each in positive and negative ion mode. Combining these 6 LC‐MS platforms, we annotated 681 unique metabolites in liver samples. The complete data are available as Supplemental Table S4.

**FIGURE 6:**
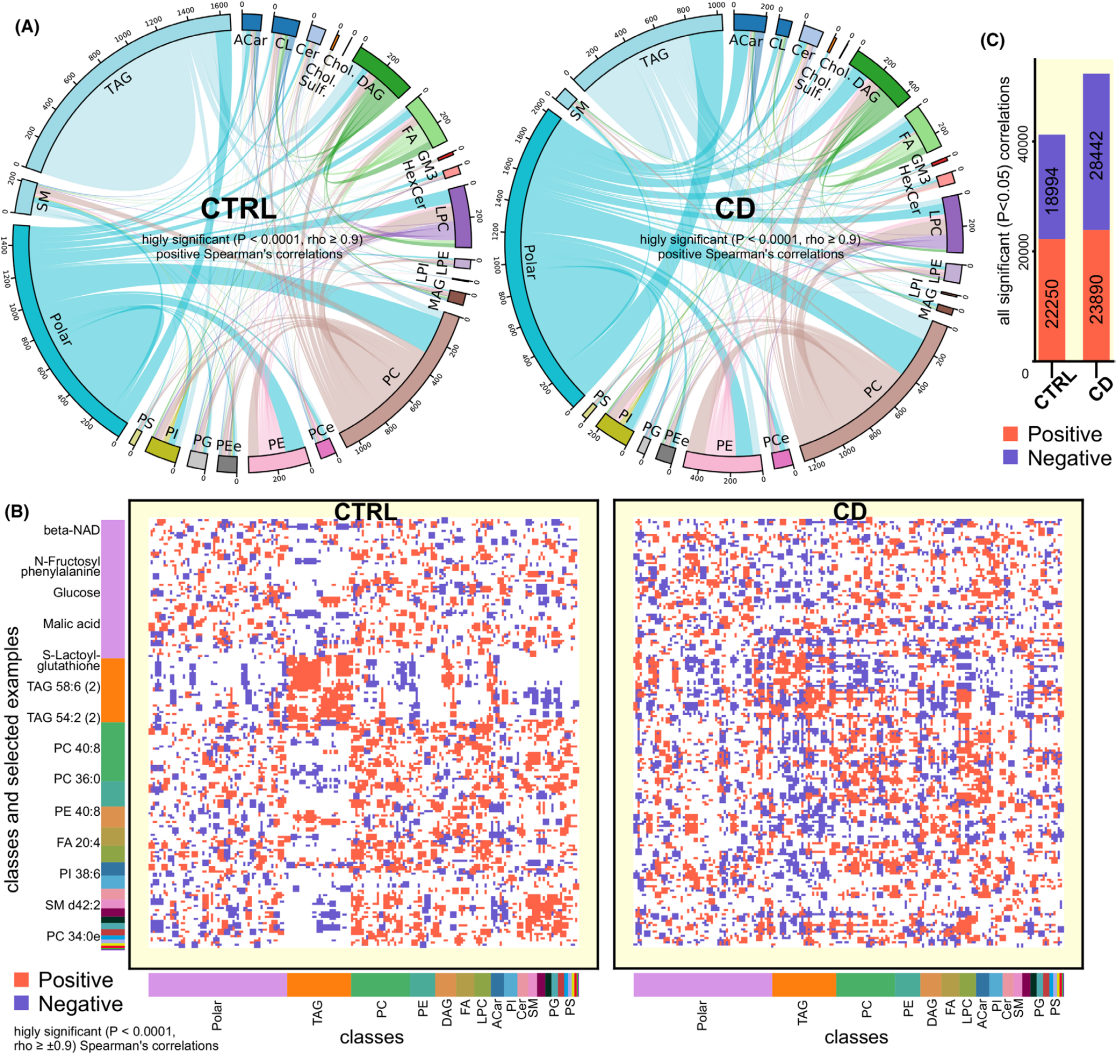
Coherence of liver metabolic pathways changes in response to CD regime. (A) Chord plots showing highly significant (*p* < 0.0001) positive correlations (with the number of individual correlations illustrated by the thickness of a ribbon) between polar metabolites and individual lipids clustered to major classes within CTRL (left) and CD (right) liver. Each link depicts Spearman's correlation with rho ≥0.9. Colored strips show Polar metabolites (amino acids, saccharides and others) and lipid classes: SM, sphingomyelins; TAG, triacylglycerols; ACar, acylcarnitines; CL, cardiolipins; Cer, ceramides; cholesterol and cholesterol sulfate, DAG, diacyglycerols; FA, fatty acids; GM3, GM3 ganglioside; HexCer, hexosylceramides; LPC, lysophosphatidylcholines; LPE, lysophosphatidylethanolamines; LPI, lysophosphatidylinositol; MAG, monoacylglycerols; PC, phosphatidylcholines; PCe, ether phosphatidylcholines; PE, phosphatidylethanolamines; PEe, ether phosphatidylethanolamines; PG, phosphatidyliglycerols; PI, phosphatidylinositols; PS, phosphatidylserines. (B) Correlation Hinton plot for all 681 detected lipids and polar metabolites in CTRL (left) and CD (right) liver. Only the most significant (|rho| ≥ 0.9; *p* < 0.0001) Spearman's correlations are depicted as either red (positive) or blue (negative) rectangle. Major classes are color coded and shown below the x‐axis with selected examples next to the y‐axis. (C) Number of all significant polar metabolite and lipid correlations (positive in red, negative in blue) in both CTRL and CD.

We first analyzed compounds that show significant correlations and may thus share metabolic pathways and function. We hypothesized, that CD regime will negatively affect the temporal organization of liver metabolome and lipidome. Interestingly, while CD disrupted correlations within some lipid classes (particularly within triacylglycerols, TAG, CD decreased the number of significant positive correlations by a factor of 3, Figure 6A), the total number of significant positive correlations slightly increased (Figure 6B,C), likely due to restricted feeding and its dominant effect on the metabolic tissues. For example, CD increased the number of positive correlations between polar metabolites (such as amino acids and sugars) and triacylglycerols or acylcarnitines (Figure 6A). On the other hand, the number of significant negative correlations increased dramatically in response to CD (Figure 6B,C), suggesting widespread reorganization of metabolic networks due to circadian disruption.

**FIGURE 7:**
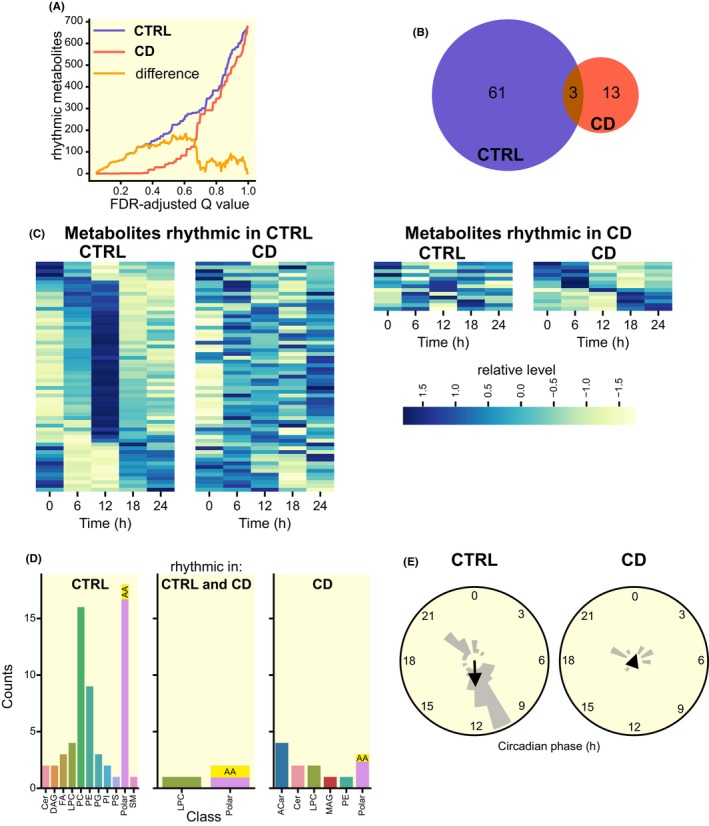
Number of significantly rhythmic polar metabolites and lipids in the liver is markedly decreased in response to CD regime. (A) The rhythmicity threshold (*Q* < 0.4) was chosen after plotting the number of rhythmic compounds in CTRL (blue) and CD (red) against FDR *Q* value of two independent detection methods (eJTK, BIO_CYCLE); yellow line shows the difference in number of rhythmic metabolites between CTRL and CD as a function of *Q*. (B) Venn diagram of compounds identified as rhythmic in CTRL (blue) and CD (red) liver samples. (C) Heatmap of compounds identified as significantly rhythmic in CTRL (left) or CD (right) liver samples using two independent detection methods (eJTK, BioCycle), normalized and sorted by phase. (D) Polar metabolites (AA, amino acids) and lipid classes (ACar, acylcarnitines; Cer, ceramides; DAG, diacyglycerols; FA, fatty acids; LPC, lysophosphatidylcholines; MAG, monoacylglycerols; PC, phosphatidylcholines; PE, phosphatidylethanolamines; PG, phosphatidyliglycerols; PI, phosphatidylinositols; PS, phosphatidylserines; polar metabolites such as amino acids and saccharides, SM, sphingomyelins) identified as rhythmic in CTRL (left), CD (right) or in both groups (middle). (E) Polar histogram of compounds identified as rhythmic in CTRL (left) and CD (right) liver samples with calculated Rayleigh vector showing the mean phase.

We next performed rhythmicity detection to study the effect of CD with temporal resolution. We used two independent methods (eJTK and BIO_CYCLE), plotted the rhythmic metabolites against the resulting false discovery rate adjusted *Q* values (Figure 7A), showing that CD dramatically reduces the number of rhythmic metabolites across the whole range of resulting *Q* values. We then selected a specific threshold (*Q* < 0.4) and analyzed the profiles by one‐way ANOVA to detect significant time variation, resulting in 61 metabolites labeled as rhythmic in CTRL and only 13 in CD, with 3 of them rhythmic under both conditions (Figure 7B,C). Interestingly, while phosphatidylcholines were the dominant rhythmic lipid class in CTRL, none of them stayed rhythmic under CD; instead, rhythmic acylcarnitines were highly overrepresented in CD and missing in CTRL (Figure 7D).

**FIGURE 8:**
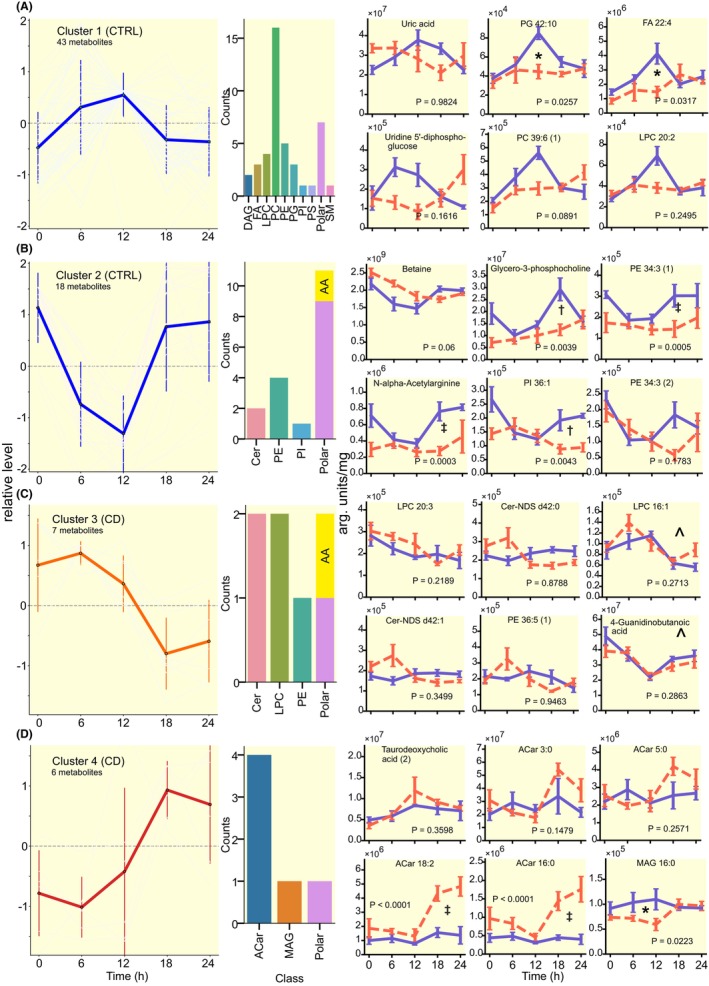
Majority of rhythmic lipids cluster at the start of locomotor activity. (A) Polar metabolites and lipids identified as rhythmic in (A and B) CTRL samples (clusters 1–2) or (C and D) CD samples (clusters 3–4) were divided to subgroups based on hierarchical K‐means clustering. Plots on the left show traces of normalized level of each rhythmic compound in each corresponding cluster. Bar plots in the middle show the lipid classes and polar metabolites enriched in each rhythmic cluster. Circadian time profiles on the right show 6 examples of polar metabolites and lipids from each rhythmic cluster, depicted in both in CTRL (blue, full line) and CD (red, dashed line) liver samples, mean ± SEM; *p* values show the result of 2 way ANOVA comparison between group; ^ depicts metabolites rhythmic in both CTRL and CD group (clusters 1 and 3). *p < 0.05, ‡*p* < 0.0001

Most rhythmic compounds in the CTRL group peaked around ZT12, but phases of rhythmic compounds in CD group were more dispersed (Figure 7C,E). K‐means clustering divided the compounds further into two distinct groups based on their phase and amplitude. In the CTRL group, 36 lipids and 7 polar metabolites had high levels during ZT6‐12 (Figure 8A) and 7 lipids and 11 polar metabolites (including 2 amino acids) had high levels between ZT18‐24 (Figure 8B). Of the 13 rhythmic metabolites identified in the CD group, 7 peaked around ZT6 (Figure 8C) and 6, including all 4 rhythmic acylcarnitine species, peaked around ZT18 (Figure 8D). A two‐way ANOVA to determine differences between CTRL and CD profiles showed that ACar 16:0 and ACar 18:2 were also significantly (*p* < 0.0001) upregulated in the CD groups, specifically between ZT18 and ZT24.

### 2.6 CD affects circadian expression of genes involved in metabolic regulation

**FIGURE 9:**
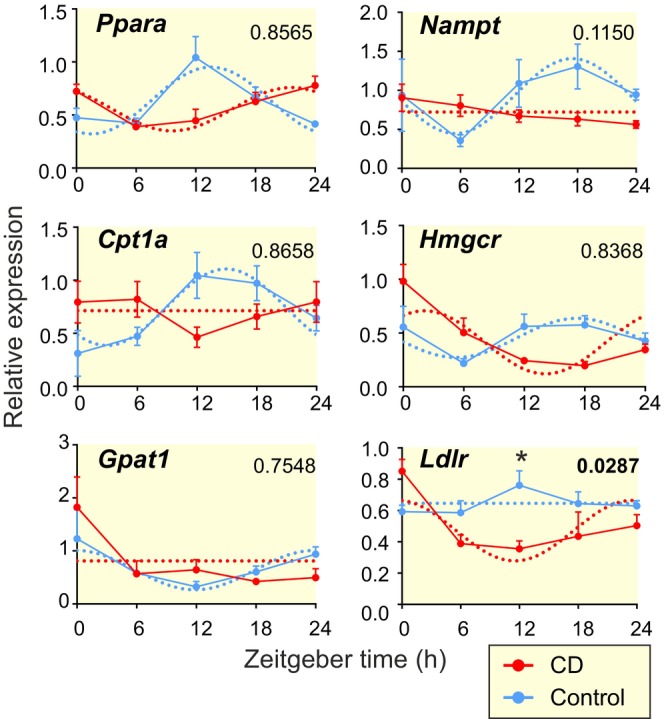
Daily mRNA profiles of selected metabolic regulatory genes in the liver measured by RT qPCR in CTRL (black) and CD (orange) group and analyzed by cosinor and two‐way ANOVA; mean ± SEM, *p < 0.05.

Since CD affected the liver lipidome, we used RT qPCR to analyze the circadian profiles of genes involved in lipid metabolism (Figure 9, Table S3). Expression of Peroxisome proliferator‐activated receptor alpha (PPAR‐α, *Ppara* gene), the main regulator of fatty acid uptake and catabolism, was highly rhythmic in CTRL and this rhythm was phase‐reversed in response to CD. PPAR‐α activates Carnitine palmitoyltransferase I (CPT1, *Cpt1a* gene), which facilitates transfer of fatty acids to mitochondria by forming ACars, a rate limiting step in beta‐oxidation. *Cpt1a* lost its rhythm in response to CD. However, this alone would not explain the observed increase of certain ACar species levels and there is likely a more widespread dysregulation of downstream PPAR targets. For example, mitochondrial glycerol‐3‐phosphate acyltransferase 1 (GPAM), encoded by the *Gpat1* gene, competes with CPT1 for acyl‐CoA substrates used in glycerolipid biosynthesis. This enzyme, which is expressed rhythmically in antiphase to *Cpt1a* in the liver, also lost its mRNA rhythm in response to CD, potentially explaining the significant loss of correlations between various TAG species. Genes involved in other metabolic pathways in the liver were also affected. Nicotinamide phosphoribosyltransferase (*Nampt* gene), the rate‐limiting enzyme in the NAD^+^ salvage pathway whose activity affects the master metabolic regulator SIRT1 and which forms a crucial feedback loop between metabolism and circadian clock, lost its mRNA rhythm in response to CD. Circadian expression of gene for HMG‐coenzyme A reductase (*Hmgcr*), rate‐limiting enzyme in the biosynthesis of cholesterol and the target of statins, was also markedly changed in response to CD, pointing to the disruption of cholesterol metabolism. Interestingly, low‐density lipoprotein receptor gene (*Ldlr*) was significantly downregulated (two‐way ANOVA, *p* = 0.0287) in response to CD, resulting in a distinct circadian‐like pattern of expression. Importantly, lower expression of *Ldlr* may lead to inefficient endocytosis of LDL and increased levels of blood cholesterol.

## 3 DISCUSSION

In real life, humans often experience irregular, low‐amplitude environmental LD cycles due to artificial lighting, and their eating behavior does not match their endogenous circadian clock. The health implications and underlying mechanisms of these misalignments are not yet sufficiently understood, mainly because these conditions are difficult to replicate in experiments with rodent models. To fill the gap, we developed a novel protocol of a complex CD in the rat that induces dampening of the rhythmic output signal from the SCN to periphery and affects hierarchy among the clocks within the circadian system needed for proper entrainment of the peripheral clocks. We analyzed impact of CD on behavioral activity, sleep patterns, gene expression, glucose metabolism and liver lipidome.

In the SCN, exposure of animals to CD did not abolish rhythmic expression of canonical clock genes, but severely dampened rhythmic expression of two of the E‐box‐regulated clock‐controlled genes, *Avp* and *Prok2*. Neurons producing arginine vasopressin (AVP) provide connection from the SCN to the hypothalamic paraventricular nucleus (PVN), which is the major pathway mediating rhythmic neuro‐humoral and autonomous nerve regulations.^49^ Prokineticin (PROK2) is a secretory peptide, which has been recognized as a messenger diffusing rhythmically from the SCN and binding to receptors in many brain regions.^20^ For example, PROK2 binding in the preoptic area is involved in regulation of estrous cycle.^50^ In accordance, CD‐attenuated PROK2‐mediated output from the SCN correlated with significant disruption of the 4‐day‐regularity of the estrous cycle‐related behavior in our rats. Additionally, PROK2 binds receptors in the DMH, which is another important hub relaying the signals from the SCN to integrate the arousal/sleep and feeding/fasting cycles.^51^, ^52^ Sensitivity of DMH oscillator to feeding/fasting cycle has been previously observed,^53^, ^54^ however, its responses in conditions of weakened or abolished SCN signaling have not been studied. Exposure to CD affected the DMH oscillator as well as the sleep patterns; the animals slept less and their sleep was more fragmented during the “inactive” phase (ZT0‐12), and the opposite was true for the “active” phase (ZT12‐24). Previously, genetic knock out of PROK2 receptors disrupted rhythms in gene expression in peripheral clocks.^55^ Our results suggest that the PROK2‐attenuated signaling from the SCN to the DMH due to CD exposure dampened and shifted the DMH oscillator, which significantly impaired the sleep/wake patterns. Indeed, worsening of sleep in our CD rats during the daytime (inactive phase) correlated with the downregulation of *Prok2* expression during ZT0–ZT12, in accordance with the fact that its peak levels are coupled with inactivity during the cycle.^20^ Altogether, our results indicate that CD impairs the SCN clock‐driven rhythmic output from the SCN to the brain preoptic area and DMH, which significantly affects their function.

In addition, the CD‐induced impairment of rhythmic output from the SCN to the brain regulatory areas significantly affected downstream peripheral clocks in the liver, colon and pancreas. All these peripheral clocks of CD rats responded to both attenuation of signals from the SCN and reversed feeding, as documented by abolition of rhythms in *Per1,2* and the phase reversal of *Nr1d1/ Bmal1* rhythms, respectively. Indeed, *Per2* was previously demonstrated to sense input signals from the SCN and integrate them into the local TTFL,^25^ whereas *Nr1d1* ^56^ and *Bmal1* ^57^ genes were found to be closely associated with changes in the local metabolic state. Intriguingly, only 2 days of rRF at the end of the CD protocol were sufficient to completely reverse the phase of the accessory *Bmal1/Nr1d1* loop of the TTFL in all three peripheral tissues (food was returned after 2 days of rRF at ZT0 on the day of sampling). This is much faster than previously shown for hepatocytes under LD12:12, which needed more than 6 days for full phase‐reversal due to daytime feeding.^58^ Importantly, the SCN lesion accelerated the resetting so that the hepatocytes attained a steady state phase shift earlier.^58^, ^59^ In accordance, we show that weakening of the SCN‐derived signaling to periphery due to CD is sufficient to accelerate entrainment of peripheral clocks by rRF. Remarkably, even the clock in pancreatic tissue shifted rapidly in CD‐exposed animals, although it required more than 10 days of rRF under LD12:12 conditions, as we had previously reported.^45^ Consequently, CD disturbed hierarchy between the central and peripheral clocks via suppressing the SCN output, which abolished the phase‐leading position of the SCN clock and prevented thus the SCN ability to entrain peripheral clocks to external LD cycle.

These results validate the CD protocol as a model of disruption of the circadian system in its complexity, which is supposed to impair regulation of physiological processes. Indeed, CD impaired glucose homeostasis, and in correlation with disturbances of the *Per1,2* rhythms in the liver, it disturbed daily metabolome and lipidome profiles in the tissue. Accordingly, genetic knock out *Per1/2* genes in mice impaired glucose tolerance^60^ and lipid metabolism was altered in *Per2−/−* mice.^61^

In our study, CD rearranged the organization of metabolic pathways in the liver. The overall number of negatively correlated lipids and polar metabolites increased by 33% under CD, while the overall number of positively correlated compounds remained similar. It is likely that the restriction of the feeding interval under CD compared to ad libitum condition caused more coherent spiking of some metabolites and lipids, compensating to some extent for the loss of rhythmic regulation of metabolic pathways. For example, liver triglycerides largely lost their coherent temporal profiles in response to CD, partly due to the decreased levels of several TAG species, and the expression of the *Gpat1* gene responsible for glycerolipid biosynthesis lost its rhythmic expression. Interestingly, a previous study showed reduced TAG levels in mice lacking *Per2* gene.^61^

Coherent temporal regulation of metabolic functions is important for liver homeostasis.^62^ We found that exposure to CD significantly reduced the number of rhythmic metabolites. Rhythm analyses confirmed significant daily rhythm in 71 (10.4%) of 681 detected lipids and polar metabolites, with 58 rhythmic selectively in the CTRL (mostly polar metabolites, phosphatidylcholines (PC), phosphatidylethanolamines (PE) and related lipid classes), but only 10 rhythmic selectively in the CD. Only three metabolites were assigned to be rhythmic in both groups. In CTRL, the most abundant cluster peaking around the switch between the day and night was mostly represented by PC (*n* = 15), while the oppositely phased cluster was dominated by polar metabolites, including amino acids (*n* = 11), demonstrating temporal separation of metabolic pathways. This separation was much less discernible under CD regime. Expression of genes for responsible enzymes (GPAM and CPT1) and their transcriptional regulator (PPARa)^63^ responded sensitively to CD, and most lipids lost their rhythmic levels. Interestingly, levels of four long‐chain acylcarnitine (ACar) species, essential intermediates in mitochondrial fatty acid oxidation, significantly elevated during ZT12‐24, pointing to a time‐specific increase of mitochondrial beta‐oxidation of fatty acids. This may be explained either by the longer fasting window during the 2‐day rRF regime possibly prioritizing lipolysis in adipocytes and subsequent beta‐oxidation of fatty acids as the energy source^64^ in the liver, or simply by the overall disruption of coherent liver metabolism due to CD, additionally reflected in the changed expression profiles of genes crucial for NAD^+^ and cholesterol metabolism. The latter possibility seems more likely because our rRF protocol with 12‐h food availability interval did not led to significant starvation, as it had no effect on body weight. In addition, previous studies did not reveal direct effect of feeding on driving oscillations in a large portion of lipids^65^ and most lipids did not oscillate during restricted feeding regime in *Per1/2* knockout mice.^66^ Our data suggest a possibility that the CD‐induced disturbance of sleep patterns may participate in the effect of CD on the metabolomics profiles and glucose homeostasis. Indeed, in humans, sleep disturbance not only increased insulin resistance but was also associated with an increase of ACar levels in plasma,^67^, ^68^ including the long‐chain ACar 18:2,^69^ which was also elevated in the liver of our rats exposed to CD. These data pointed to ACars as, a potential mechanistic link between sleep disturbance and adverse metabolic effects.^69^ They support the role of sleep disruption rather than feeding/fasting rhythm in the CD‐modulated ACar profiles and related increase in glucose tolerance in our rats.

The major weakness of our study is the limited number of female rats available, which necessitated a sparse 6‐h sampling interval over the 24‐h period and resulted in lower confidence of rhythm detection and phase determination for the metabolome and lipidome profiles. To minimize the number of false positives, we used a relatively stringent threshold for rhythmicity detection. However, the sampling intervals and number of animals per time point were sufficient for the reliable detection of rhythmicity in the gene expression profiles, as shown by the sufficiently high R^2^ of the cosine analysis for most profiles. Most previous studies on the effects of circadian rhythm disruption on behavior and daily metabolome and lipidome profiles have used male mice. This is one of the first studies to focus on female rats. It validates a novel CD protocol that aims to uncouple the central and peripheral circadian clocks.

## 4 MATERIALS AND METHODS

### 4.1 Ethical statement

The experimental protocol was approved by the Animal Care and Use Committee of the Institute of Physiology and was in accordance with the Animal Protection Law of the Czech Republic as well as the European Community Council directives 2010/63/EU.

### 4.2 Animals and experimental protocol

The female Wistar rats (Institute of Physiology, Czech Academy of Sciences, Prague) were housed individually since weaning under 12‐h light/12‐h darkness (LD12:12) with lights on at 06:00 and off at 18:00. Standard chow diet (calories from protein 24%, fat 11% and carbohydrates 65%) and drinking water were available ad libitum. At age of 6 weeks, the rats were randomly divided into two groups: (1) control animals (CTRL; *n* = 23) were maintained in the same conditions with LD12:12 with ad libitum food access throughout the experiment, (2) animals exposed to an experimental protocol to disrupt the circadian system (complex chronodisruption, CD; *n* = 26) which lasted 4 weeks. The protocol consisted of 4 weekly cycles, each of which consisted of constant light with food ad libitum for 5 days, followed by reversed restricted feeding (rRF) with food access restricted to the light phase of the original LD12:12 (from 09:00 till 15:00) for 2 days. At the end of the protocol, animals of both groups were sacrificed in 6‐h intervals (*n* = 4–5 per time point) over 24 h by rapid decapitation under deep terminal anesthesia (i.p. sodium thiopental 50 mg/kg b.w.). CTRL animals were sacrificed under LD12:12, and CD animals were sacrificed at the same time points after completing the last weekly cycle on the first day in constant light (see Figure 1A for experimental flowchart).

Throughout the study, locomotor activity was continuously monitored. Additionally, body weight and weight of the consumed food were recorded once a week. The intraperitoneal glucose tolerance test (IPGTT) was performed during the last week of the experimental protocol.

### 4.3 Locomotor activity monitoring

Locomotor activity was monitored as described previously.^70^ Briefly, infrared movement detectors were placed in the middle of the cage top to record the animals' movement every minute. The readings were further fed to the detector (Dr. H.M. Cooper, INSERM, France) and double‐plotted actograms were generated for visualization of data. The resulting data, including calculations of the chi‐square periodograms with *p* < 0.001, were analyzed using ClockLab toolbox (Actimetrics, Illinois).

The sleep parameters were assessed by Actiwatch Activity and Sleep Analysis V 5.42 software (Cambridge Neurotechnology Ltd., Cambridge, UK), as described previously.^71^ The data from the last 4 days of the protocol were analyzed in order to detect an actual impact of the chronic intervention on behavior at the time of sampling the animals. The analyzed parameters were inactivity (as a correlate of sleep) assessed during the 24 h interval, as well as percent of inactive time and fragmentation index during the dark and light phases of the actual LD12:12 cycle. The data were averaged for each animal. Finally, the average values of these parameters (total inactivity during the 24 h, percent of immobile minutes and fragmentation index during the dark and light phases) were calculated for each group (CTRL, *n* = 23; CD, *n* = 26; mean ± SD).

### 4.4 Intraperitoneal glucose tolerance test (IPGTT)

On the 3rd day of the last week of the CD protocol, rats were fasted for 3 h (starting from the lights on) and the IPGTT was performed at 09.00 h. Glucose was measured using a glucose meter (GlucoLab, Infopia Co., Ltd., Korea) in blood drop collected from the tail as previously published.^72^ Glucose levels were detected before the glucose injection (basal levels). Then, glucose was injected (2 g/kg b.w., i.p.) and blood glucose levels were detected 15, 30, 60, 120, and 180 min after the injection. The incremental area under the curve (AUC) was calculated.

### 4.5 Tissue sampling

The brains were isolated, snap‐frozen on dry ice, and kept at −80°C. Frozen brains were sectioned using cryostat into 25 μm coronal sections, and tissue samples containing the SCN, dorsomedial hypothalamus (DMH), and hippocampus (HPC) were dissected using a laser microdissector (LMD6000, Leica). The samples were stored in RNAlater (Qiagen, Valencia, CA, USA) until RNA isolation. Samples of liver, pancreas, and colon were collected as previously described.^72^ The distal colon was excised and rinsed with phosphate‐buffered saline. A longitudinal cut was made and the epithelial layer was gently scraped off. The epithelial layer was stored in RNAlater at −20°C until the isolation of RNA.

### 4.6 RNA isolation and real‐time RT qPCR

Total RNA from the brain regions and peripheral tissues were isolated using RNeasy Microkit (Qiagen, Valencia, USA) as per manufacturer's instructions and reverse‐transcribed into cDNA immediately using High capacity cDNA Reverse Transcription Kit (ThermoFischer Scientific; Waltham, MA, USA). The diluted cDNA samples from dissected brain regions were amplified using LightCycler480 Real‐Time PCR System (LC480, Roche, Basel, Switzerland) in 14‐μl reactions using 5x HOT FIREPol Probe qPCR mix Plus (Solis Biodyne; Tartu, Estonia) and TaqMan Gene Expression Assays (Life Technologies; San Francisco, CA, USA) spanning exon junctions specific for rat genes *Bmal1* (Rn00577590_m1, FAM‐labeled), *Per1* (Rn01325256_m1, FAM), *Per2* (Rn01427704_m1, FAM), and *Nr1d1* (Rn01460662_m1, FAM). The diluted cDNA samples from peripheral tissues and SCN were amplified on LC480 using Xceed SybrGreen qPCR mastermix with predesigned forward and reverse primers (KiCqStart® SYBR® Green) by Sigma (listed in the Table S1). Relative quantification was achieved using ΔΔCT method^73^ by normalization to β2‐microglobulin (*B2m*) for the SCN, *B2m* and ribosomal protein S18 (*Rps18*) for DMH, liver and colon, and *Rps18* and TATA binding protein (*Tbp*) for the pancreas.

### 4.7 Metabolomic and lipidomic profiling of the liver

Metabolites were extracted from liver samples (20 mg) using bi‐phase extraction with methanol, methyl *tert*‐butyl ether, and 10% water, as detailed in Supplementary Methods. Lipidomics and metabolomics were performed on a liquid chromatography‐mass spectrometry (LC–MS) system consisting of a Vanquish UHPLC System (Thermo Fisher Scientific, Bremen, Germany) coupled to a Q Exactive Plus mass spectrometer (Thermo Fisher Scientific, Bremen, Germany),^74^ followed by processing of raw instrumental files using MS‐DIAL.^75^ For a detailed description of the LC process, use MS parameters, quality control, and data processing; see Supplementary Methods. Absolute values (peak heights) of 681 identified metabolites were further analyzed; no outliers were removed. Differences in average levels between metabolites in both groups were analyzed by DESeq2 using RNAlysis 3.9 GUI.^76^ To identify potentially rhythmic metabolites, three combined approaches were employed. First, one‐way ANOVA was used to identify metabolites with significant (*p* < 0.05) variation across time points. Two rhythmicity detection algorithms were then used in succession—eJTK (https://biodare2.ed.ac.uk/),^77^ and BIO_CYCLE (http://circadiomics.igb.uci.edu/biocycle).^78^ After examining the dependence of number of rhythmic metabolizes on Benjamini–Hochberg (BH) false discovery rate (FDR)‐adjusted score (FDR corrected empirical *p* value in case of eJTK, FDR adjusted *Q* value in case of BIO_CYCLE) of both algorithms, the rhythmicity threshold was set at below 0.4. To detect the phase of the cycling metabolites, BIO_CYCLE variable LAG was used. Rhythmic metabolites in each group were then clustered according to gap‐statistics by K‐means clustering in RNAlysis. Plots were prepared in either Prism 8 (Graphpad, USA), or seaborn + matplotlib and statistical comparisons (one‐way and two‐way ANOVA, Mann–Whitney test, Wilcoxon signed rank test) were performed in Prism, or Python packages scipy and statsmodels.

### 4.8 Statistical analysis

Data are presented in accordance with good publishing practice in physiology.^79^ Statistical analyses of activity and gene expression profiles were performed in Prism, *p* < 0.05 was required for significance. Data is presented as means ± SD. The two‐way ANOVA with Sidak's multiple comparisons test for post hoc analysis was used to analyze effect of group for the locomotor activity data, effect of time and group (CTRL and CD) for glucose levels in IPGTT and the daily gene expression. The unpaired *t*‐test was used to analyze the difference in activity parameter, food intake, basal glucose levels and AUC between the groups. To confirm presence or absence of a circadian rhythm in the gene expression, significant fit by cosine analysis were required. Cosine analysis was performed as previously described.^70^ Briefly, two alternative regression models to differentiate between rhythmic and non‐rhythmic expression were used: either a horizontal straight line (null hypothesis) or a single cosine curve (alternative hypothesis), defined by the equation Y = mesor + (amplitude*cos(2*π* (X − acrophase)/wavelength)) with a constant wavelength of 24 h. The extra sum‐of‐squares F test was used for comparison, and the cosine curve parameters were calculated if the *p* value exceeded 0.05.

## AUTHOR CONTRIBUTIONS

**Shiyana Arora:** Investigation; writing – original draft; formal analysis. **Pavel Houdek:** Investigation; formal analysis; visualization. **Tomáš Čajka:** Data curation; methodology; formal analysis; writing – review and editing. **Tereza Dočkal:** Investigation. **Martin Sládek:** Data curation; methodology; formal analysis; visualization; writing – review and editing. **Alena Sumová:** Conceptualization; funding acquisition; writing – original draft; writing – review and editing; project administration; supervision; validation.

## FUNDING INFORMATION

The project no. LX22NPO5104 is funded by the European Union Next Generation EU and the Research Project RVO: 67985823.

## CONFLICT OF INTEREST STATEMENT

The authors declare no competing interests.

## DECLARATION OF GENERATIVE AI AND AI‐ASSISTED TECHNOLOGIES IN THE WRITING PROCESS

The authors declare assistance of no AI technologies.

## Supporting information


Figure S1.

Table S1.

Table S2.

Table S3.

Table S4.

Methods S1.

## Data Availability

The data that supports the findings of this study are available in the supplementary material of this article.
