# Supplementary material for: Chronodisruption that dampens output of the central clock abolishes rhythms in metabolome profiles and elevates acylcarnitine levels in the liver of female rats
Source: Acta Physiol (Oxf). 2025 Jan 13;241(2):e14278. doi: 10.1111/apha.14278 (PMC11726269; doi:10.1111/apha.14278)

Supplementary Figure S1. Individual activity records (actograms) of animals maintained in LD12:12 (Control group) or exposed to the chronodisruption protocol (CD). Each line represents one day of the experiment. For details, see Materials and Methods.

Control group

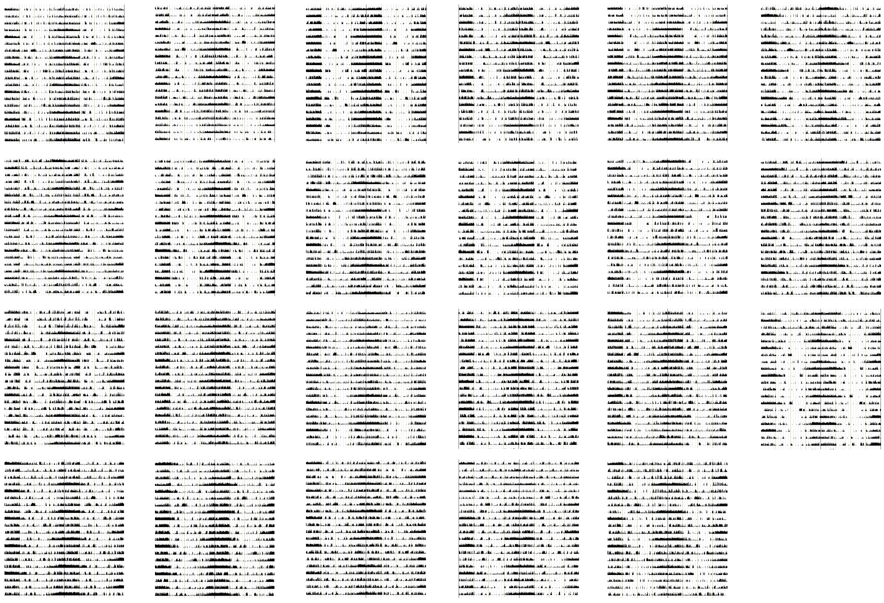

CD (chronodisruption) group

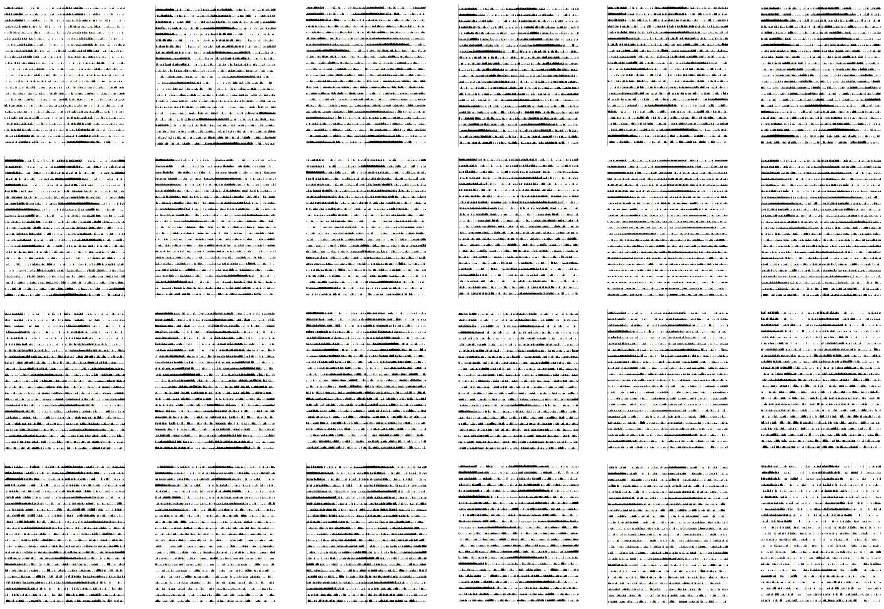

Supplement: Supplementary file 1 — Figure S1. [file APHA-241-e14278-s002.pdf]
