# Supplementary material for: Chronodisruption that dampens output of the central clock abolishes rhythms in metabolome profiles and elevates acylcarnitine levels in the liver of female rats
Source: Acta Physiol (Oxf). 2025 Jan 13;241(2):e14278. doi: 10.1111/apha.14278 (PMC11726269; doi:10.1111/apha.14278)
Supplement: Supplementary file 2 — Table S1. [file APHA-241-e14278-s005.docx]

**Supplementary Table S1**. Sequences of primers used for RT-qPCR assay

| **Rat primers** | |
| --- | --- |
| primer | sequence |
| *Tbp* | F-CATCATGAGAATAAGAGAGCC R-GGATTGTTCTTCACTCTTGG |
| *Rps18* | F-ACTGCCATTAAGGGTGTG R-GTCAGGGATCTTGTATTGTC |
| *B2m* | F-CGCTCGGTGACCGTGATCTTTCTG R-TGAGGTGGGTGGAACTGAGACACG |
| *Per2* | F-GAATTTTCACAACAACCCAC R-TGTAGGATCTTCTTGTGGATG |
| *Per1* | F-GCACTTCGGGAGCTCAAACTTC R-TCCATGGCACAGGGCTCACC |
| *Arntl* | F- ATGAAAACATTGAGAGGTGC R-GGATCTTGAAGACAGATTCG |
| *Nr1d1* | F-GCTGTGCGGGAGGTGGTAGAAT R-TGTAGGTTGTGCGGCTCAGGAA |
| *Cry1* | F-GTGGTGGCGGAAACTGCTCTC R-ACTCTGTGCGTCCTCTTCCTGA |
| *Dbp* | F-GCTAATGACCTTTGAACCTG R-AGTACTTCTCATCCTTCTGTTC |
| *Avp* | F-TCAACACTACGCTCTCTG R-CTGTCTCAGCTCCATGTC |
| *Vip* | F-TTAGAAAGCAAATGGCTGTG  R-CAAGAATTCTCTGATCTTCAGC |
| *Prok2* | F-GACTCGGAAAAGTCATGTTG R-CAAAATGGAACCTCCTTCTTC |
| *Ppara* | F-CTGCTATAATTTGCTGTGGAG R-GAGTTTTGGGAAGAGAAAGG |
| *Nampt* | F-CTTTGGTTCTGGTGGCGCTTTGCTAC  R-GCCGGCCCTTTTTCGACCTTTTGTT |
| *Cpta1* | F-GTTCATCCGGTTCAAGAATG  R-CTCACAATGTTCTTCGTCTG |
| *Hmgrc* | F-CAAGATGATCATGTCTTTAGGC  R-GTAAAACTGCCAGAGAGAAAC |
| *Gpat1* | F-CCATCTTCAGTACCTTGATTC  R-AGGAGTTCAACTATATGCCC |
| *Hnf4a* | F-TGTGTGAGTCTATGAAGGAG R-ATGTAGTCATTGCCTAGGAG |
| *Pgc1* | F-CAGTTTCATTCGACCTGCGTAA  R-CCGTAAATCTGCGGGATGATG |
| *Ldrl1* | F-CAGTGTGAAGATATTGACGAG  R-TCATCTTACGTACCTCATGG |
