# Supplementary material for: Chronodisruption that dampens output of the central clock abolishes rhythms in metabolome profiles and elevates acylcarnitine levels in the liver of female rats
Source: Acta Physiol (Oxf). 2025 Jan 13;241(2):e14278. doi: 10.1111/apha.14278 (PMC11726269; doi:10.1111/apha.14278)
Supplement: Supplementary file 3 — Table S2. [file APHA-241-e14278-s004.docx]

**Supplementary Table S2**. Results of cosinor analyses of the daily profiles of clock gene expression in the suprachiasmatic nuclei (SCN), the dorsomedial hypothalamus (DHM), and three peripheral tissues (liver, colon, pancreas) of control mice (CTRL) and mice exposed to chronodisruption protocol (CD). Mean expression levels (mesor), peak of the rhythm (acrophase), and amplitude are expressed as the mean ± S.E.M. R^2^ (goodness of fit) and statistical significance (P) are depicted. Data in red highlight data for profiles that were rhythmic in CTRL group and lost rhythmicity in CD group.

|  |  | ***Per1*** | | ***Per2*** | | ***Nr1d1*** | | ***Bmal1*** | |
| --- | --- | --- | --- | --- | --- | --- | --- | --- | --- |
| **SCN** |  | CTRL | CD | CTRL | CD | CTRL | CD | CTRL | CD |
|  | Mesor  ±SEM | 0.65  ±0.05 | 0.66  ±0.07 | 0.12  ±0.01 | 0.19  ±0.01 | 0.41  ±0.05 | 0.55  ±0.05 | 0.80  ±0.04 | 1.07  ±0.04 |
|  | Acrophase  ±SEM | 4.11  ±0.65 | 23.67  ±1.0 | 9.43  ±0.61 | 10.58  ±0.75 | 2.30  ±0.41 | 22.98  ±0.69 | 18.20  ±0.75 | 19.11  ±0.74 |
|  | Amplitude  ±SEM | 0.43  ±0.08 | 0.40  ±0.09 | 0.08  ±0.01 | 0.06  ±0.01 | 0.24  ±0.02 | 0.3  ±0.07 | 0.28  ±0.07 | 0.42  ±0.09 |
|  | R^2^ | 0.5843 | 0.4577 | 0.6730 | 0.6068 | 0.8334 | 0.6498 | 0.4709 | 0.5073 |
|  | P | **0.0002** | **0.0016** | **<0.0001** | **<0.0001** | **<0.0001** | **<0.0001** | **0.0017** | **0.0006** |
|  |  |  |  |  |  |  |  |  |  |
| **DMH** |  | CTRL | CD | CTRL | CD | CTRL | CD | CTRL | CD |
|  | Mesor  ±SEM | 0.42  ±0.04 | 0.39  ±0.04 | 0.77  ±0.06 | 0.77  ±0.05 | 0.64  ±0.03 | 0.70  ±0.05 | 13.41  ±0.96 | 7.48  ±1.92 |
|  | Acrophase  ±SEM | --------- | -------- | --------- | 0.40  ±1.5 | 8.40  ±0.7 | 20.4  ±1.28 | 4.21  ±0.09 | --------- |
|  | Amplitude  ±SEM | --------- | -------- | --------- | 0.18  ±0.06 | 0.25  ±0.05 | 0.20  ±0.07 | 5.21  ±1.5 | --------- |
|  | R^2^ | --------- | -------- | --------- | 0.3145 | 0.5915 | 0.2963 | 0.3990 | --------- |
|  | P | 0.2059 | 0.0893 | 0.3834 | **0.0229** | **0.0002** | **0.0355** | **0.0079** | 0.8132 |
| **Peripheral tissues** | | | | | | | | | |
| **Liver** |  | CTRL | CD | CTRL | CD | CTRL | CD | CTRL | CD |
|  | Mesor  ±SEM | 0.80  ±0.07 | 0.75  ±0.1 | 0.63  ±0.07 | 0.57  ±0.08 | 0.73  ±0.12 | 0.57  ±0.14 | 14.94  ±2.84 | 11.35  ±1.17 |
|  | Acrophase  ±SEM | 13.35  ±0.93 | -------- | 20.39  ±0.06 | --------- | 11.28  ±0.08 | 22.39  ±1.09 | 1.16  ±0.74 | 13.68  ±0.07 |
|  | Amplitude  ±SEM | 0.46  ±0.10 | -------- | 0.58  ±0.10 | --------- | 0.91  ±0.16 | 0.74  ±0.19 | 8.80  ±1.58 | 17.84  ±3.81 |
|  | R^2^ | 0.5365 | -------- | 0.6809 | --------- | 0.6422 | 0.4486 | 0.5515 | 0.6232 |
|  | P | **0.0015** | 0.1095 | **<0.0001** | 0.0868 | **<0.0001** | **0.0035** | **<0.0001** | **0.0007** |
| **Colon** |  | CTRL | CD | CTRL | CD | CTRL | CD | CTRL | CD |
|  | Mesor  ±SEM | 0.78  ±0.06 | 0.70  ±0.07 | 0.71  ±0.03 | 0.73  ±0.05 | 0.82  ±0.06 | 0.62  ±0.07 | 3.54  ±0.39 | 3.15  ±0.25 |
|  | Acrophase  ±SEM | 15.18  ±0.64 | -------- | 19.45  ±0.27 | --------- | 12.41  ±0.38 | 22.46  ±0.96 | 2.85  ±0.58 | 13.31  ±0.27 |
|  | Amplitude  ±SEM | 0.47  ±0.08 | -------- | 0.57  ±0.05 | --------- | 0.87  ±0.07 | 0.38  ±0.08 | 3.69  ±0.56 | 1.85  ±0.33 |
|  | R^2^ | 0.6345 | -------- | 0.8897 | --------- | 0.8827 | 0.4789 | 0.0818 | 0.4650 |
|  | P | **<0.0001** | 0.1253 | **<0.0001** | 0.1253 | **<0.0001** | 0.0011 | **<0.0001** | **<0.0001** |
| **Pancreas** |  | CTRL | CD | CTRL | CD | CTRL | CD | CTRL | CD |
|  | Mesor  ±SEM | 0.92  ±0.12 | 1.02  ±0.26 | 1.09  ±0.11 | 0.86  ±0.1 | 16.03  ±3.94 | 8.36  ±2.64 | 11.85  ±1.58 | 11.81  ±1.68 |
|  | Acrophase  ±SEM | --------- | -------- | 19.11  ±0.69 | --------- | 11.92  ±0.79 | 22.44  ±1.29 | 1.44  ±0.72 | 13.84  ±1.02 |
|  | Amplitude  ±SEM | --------- | -------- | 0.80  ±0.15 | --------- | 27.24  ±5.44 | 11.43  ±3.59 | 12.0  ±2.20 | 9.18  ±2.30 |
|  | R^2^ | --------- | -------- | 0.6088 | --------- | 0.5824 | 0.3385 | 0.6244 | 0.4459 |
|  | P | 0.2019 | 0.2501 | **0.0002** | 0.6633 | **0.0004** | **0.0160** | **0.0001** | **0.0027** |
