# Supplementary material for: Chronodisruption that dampens output of the central clock abolishes rhythms in metabolome profiles and elevates acylcarnitine levels in the liver of female rats
Source: Acta Physiol (Oxf). 2025 Jan 13;241(2):e14278. doi: 10.1111/apha.14278 (PMC11726269; doi:10.1111/apha.14278)
Supplement: Supplementary file 6 — Methods S1. [file APHA-241-e14278-s003.docx]

**Samples extraction.** Liver samples (20 mg) were homogenized with 275 µL methanol containing internal standards (PE 17:0/17:0, PG 17:0/17:0, LPC 17:1, Sphingosine d17:1, Cer d18:1/17:0, SM d18:1/17:0, PC 15:0/18:1-d7, cholesterol-d7, TG 17:0/17:1/17:0-d5, DG 12:0/12:0/0:0, DG 18:1/2:0/0:0, LPE 17:1, oleic acid-d9, PI 15:0/18:1-d7, MG 17:0/0:0/0:0, PS 17:0/17:0, HexCer d18:1/17:0, DG 18:1/0:0/18:1-d5, TG 20:0/20:1/20:0-d5, LPG 17:1, LPS 17:1, cardiolipin 16:0/16:0/16:0/16:0) and 275 µL 10% methanol containing internal standards (caffeine-d9, acetylcholine-d4, creatinine-d3, choline-d9, TMAO-d9, N-methylnicotinamide-d4, betaine-d9, butyrobetaine-d9, creatine-d3, cotinine-d3, glucose-d7, succinic acid-d4, metformin-d6, alanine-d4, arginine-d7, aspartic acid-d3, citrulline-d2, glutamic acid-d5, glycine-13C2-15N, leucine-d3, methionine-d3, ornithine-d6, phenylalanine-d5, proline-d7, tyrosine-d4, valine-d8, carnitine-d9, C2-carnitine-d3, C3-carnitine-d3, C4-carnitine-d3, C5-carnitine-d9, C5DC-carnitine-d6, C6-carnitine-d3, C8-carnitine-d3, C10-carnitine-d3, C12-carnitine-d3, C14-carnitine-d3, C16-carnitine-d3, C18-carnitine-d3) for 1.5 min using a grinder (MM400, Retsch, Germany). Then, 1 mL of MTBE with internal standard (CE 22:1) was added, the tubes were shaken for 1 min and centrifuge at 16,000 rpm for 5 min.

For lipidomic profiling, 100 µL of upper organic phase was collected, evaporated and resuspended using 250 µL methanol with internal standard (CUDA), shaken for 30 s, centrifuged at 16,000 rpm for 5 min and used for LC-MS analysis. An aliquot of 70 µL of bottom aqueous phase was collected, evaporated, resuspended in 70 µL of an acetonitrile/water (4:1, v/v) mixture with internal standards (CUDA and Val-Tyr-Val), shaken for 30 s, centrifuged at 16,000 rpm for 5 min and analyzed using HILIC metabolomics platform. Another 70 µL of bottom aqueous phase was mixed with 210 µL of an isopropanol/acetonitrile (1:1, v/v) mixture, shaken for 30 s, centrifuged at 16,000 rpm for 5 min, and the supernatant was evaporated, resuspended in 5% methanol/0.2% formic acid with internal standards (CUDA and Val-Tyr-Val), shaken for 30 s, centrifuged at 16,000 rpm for 5 min and analyzed using HSS T3 metabolomics platform.

**LC-MS-based lipidomics**. The LC-MS systems consisted of a Vanquish UHPLC System (Thermo Fisher Scientific, Bremen, Germany) coupled to a Q Exactive Plus mass spectrometer (Thermo Fisher Scientific, Bremen, Germany).

Lipids were separated on an Acquity UPLC BEH C18 column (50 × 2.1 mm; 1.7 μm) coupled to an Acquity UPLC BEH C18 VanGuard pre-column (5 × 2.1 mm; 1.7 μm) (Waters, Milford, MA, USA). The column was maintained at 65°C at a flow-rate of 0.6 mL/min. For LC–ESI(+)-MS analysis, the mobile phase consisted of (A) 60:40 (v/v) acetonitrile:water with ammonium formate (10 mM) and formic acid (0.1%) and (B) 90:10:0.1 (v/v/v) isopropanol:acetonitrile:water with ammonium formate (10 mM) and formic acid (0.1%). For LC–ESI(−)-MS analysis, the composition of the solvent mixtures were the same with the exception of the addition of ammonium acetate (10 mM) and acetic acid (0.1%) as mobile-phase modifiers. Separation was conducted under the following gradient for LC–ESI(+)-MS: 0 min 15% (B); 0–1 min 30% (B); 1–1.3 min from 30% to 48% (B); 1.3–5.5 min from 48% to 82% (B); 5.5–5.8 min from 82% to 99% (B); 5.8–6 min 99% (B); 6–6.1 min from 99% to 15% (B); 6.1–7.5 min 15% (B). For LC–ESI(−)-MS, the following gradient was used: 0 min 15% (B); 0–1 min 30% (B); 1–1.3 min from 30% to 48% (B); 1.3–4.8 min from 48% to 76% (B); 4.8–4.9 min from 76% to 99% (B); 4.9–5.3 min 99% (B); 5.3–5.4 min from 99% to 15% (B); 5.4–6.8 min 15% (B). A sample volume of 2 and 3 μL was used for the injection in ESI(+) and ESI(–), respectively. Sample temperature was maintained at 4°C.

The ESI source and MS parameters were: sheath gas pressure, 60 arbitrary units; aux gas flow, 25 arbitrary units; sweep gas flow, 2 arbitrary units; capillary temperature, 300°C; aux gas heater temperature, 370°C; MS1 mass range, m/z 200–1700; MS1 resolving power, 35,000 FWHM (m/z 200); number of data-dependent scans per cycle, 3; MS/MS resolving power, 17,500 FWHM (m/z 200). For ESI(+), a spray voltage of 3.6 kV and normalized collision energy of 20% was used while for ESI(−) a spray voltage of −3.0 kV and normalized collision energy of 10, 20 and 30% were set-up.

**LC-MS-based metabolomics**. Polar metabolites were separated on an Acquity UPLC BEH Amide column (50 × 2.1 mm; 1.7 μm) coupled to an Acquity UPLC BEH Amide VanGuard pre-column (5 × 2.1 mm; 1.7 μm) (Waters, Milford, MA, USA). The column was maintained at 45°C at a flow-rate of 0.4 mL/min. The mobile phase consisted of (A) water with ammonium formate (10 mM) and formic acid (0.125%) and (B) acetonitrile:water (95/5) with ammonium formate (10 mM) and formic acid (0.125%). Separation was conducted under the following gradient: 0 min 100% (B); 0–1 min 100% (B); 1–3.9 min from 100% to 70% (B); 3.9–5.1 min from 70% to 30% (B); 5.1–6.4 min from 30% to 100%(B); 6.4–8.0 min 100% (B). A sample volume of 0.3 and 1 μL was used for the injection in ESI(+) and ESI(–), respectively.

Polar metabolites were also separated on an Acquity UPLC HSS T3 column (50 × 2.1 mm; 1.7 μm) coupled to an Acquity UPLC HSS T3 VanGuard pre-column (5 × 2.1 mm; 1.7 μm) (Waters, Milford, MA, USA). The column was maintained at 45°C using a ramped flow-rate. The mobile phase consisted of (A) water with formic acid (0.2%) and (B) methanol with formic acid (0.1%). Separation was conducted under the following gradient: 0 min 1% (B) 0.3 mL/min; 0–0.5 min 1% (B) 0.3 mL/min; 0.5–2 min from 1% to 60% (B) 0.3 mL/min; 2–2.3 min from 60% to 95% (B) from 0.3 mL/min to 0.5 mL/min; 2.3–3.0 min 95% (B) 0.5 mL/min; 3.0–3.1 min from 95% to 1% (B) 0.5 mL/min; 3.1–4.5 min 1% (B) 0.5 mL/min; 4.5–4.6 min 1% (B) from 0.5 mL/min to 0.3 mL/min; 4.6–5.5 min 1% (B) 0.3 mL/min. A sample volume of 5 μL was used for the injection in ESI(+) and ESI(–). Sample temperature was maintained at 4°C.

The ESI source and MS parameters were: sheath gas pressure, 50 arbitrary units; aux gas flow, 13 arbitrary units; sweep gas flow, 3 arbitrary units; capillary temperature, 260°C; aux gas heater temperature, 425°C; MS1 mass range, m/z 60–900; MS1 resolving power, 35,000 FWHM (m/z 200); number of data-dependent scans per cycle, 3; MS/MS resolving power, 17,500 FWHM (m/z 200). A spray voltage of 3.6 kV and −2.5 kV for ESI(+) and ESI(–), respectively, was used. For all metabolomics platforms a normalized collision energy of 20, 30 and 40% was used.

**Quality control**. Quality control was assured by (i) randomization of the actual samples within the sequence, (ii) injection of quality control (QC) pool samples at the beginning and the end of the sequence and between each 10 actual samples, (iii) analysis of procedure blanks, (iv) serial dilution of QC sample (0, 1/16, 1/8, 1/4, 1/2, 1), (v) checking the peak shape and the intensity of spiked internal standards and the internal standard added prior to injection.

**Data processing**. LC-MS data from metabolomic and lipidomic profiling were processed through MS-DIAL v. 3.80 software. Metabolites were annotated using in-house retention time–m/z library and using MS/MS libraries available from commercial and open sources (NIST17, MassBank, MoNA). Lipids were annotated using LipidBlast in-built in MS-DIAL [1]. Raw data were filtered using blank samples, serial dilution samples, and QC pool samples with relative standard deviation (RSD) <30%, and then normalized using locally estimated scatterplot smoothing (LOESS) approach by means of QC pool samples injected regularly between 10 actual samples.
